# Individuals with higher metabolic rates have lower levels of reactive oxygen species *in vivo*

**DOI:** 10.1098/rsbl.2015.0538

**Published:** 2015-09

**Authors:** Karine Salin, Sonya K. Auer, Agata M. Rudolf, Graeme J. Anderson, Andrew G. Cairns, William Mullen, Richard C. Hartley, Colin Selman, Neil B. Metcalfe

**Affiliations:** 1 Institute of Biodiversity, Animal Health and Comparative Medicine, University of Glasgow, Glasgow, UK; 2 School of Chemistry, University of Glasgow, Glasgow, UK; 3 Institute of Cardiovascular and Medical Sciences, University of Glasgow, Glasgow, UK

**Keywords:** fish, inter-individual variation, MitoP/MitoB ratio, oxidative stress, oxygen consumption

## Abstract

There is increasing interest in the effect of energy metabolism on oxidative stress, but much ambiguity over the relationship between the rate of oxygen consumption and the generation of reactive oxygen species (ROS). Production of ROS (such as hydrogen peroxide, H_2_O_2_) in the mitochondria is primarily inferred indirectly from measurements *in vitro*, which may not reflect actual ROS production in living animals. Here, we measured *in vivo* H_2_O_2_ content using the recently developed MitoB probe that becomes concentrated in the mitochondria of living organisms, where it is converted by H_2_O_2_ into an alternative form termed MitoP; the ratio of MitoP/MitoB indicates the level of mitochondrial H_2_O_2_ *in vivo*. Using the brown trout *Salmo trutta*, we tested whether this measurement of *in vivo* H_2_O_2_ content over a 24 h-period was related to interindividual variation in standard metabolic rate (SMR)*.* We showed that the H_2_O_2_ content varied up to 26-fold among fish of the same age and under identical environmental conditions and nutritional states. Interindividual variation in H_2_O_2_ content was unrelated to mitochondrial density but was significantly associated with SMR: fish with a higher mass-independent SMR had a lower level of H_2_O_2_. The mechanism underlying this observed relationship between SMR and *in vivo* H_2_O_2_ content requires further investigation, but may implicate mitochondrial uncoupling which can simultaneously increase SMR but reduce ROS production. To our knowledge, this is the first study in living organisms to show that individuals with higher oxygen consumption rates can actually have lower levels of H_2_O_2_.

## 1. Introduction

Oxidative stress occurs when the generation of reactive oxygen species (ROS) exceeds the capacity of antioxidant defence and repair mechanisms, thereby generating oxidative damage to lipids, DNA and proteins [1]. Given their potential role in cellular senescence, ROS are proposed as being one of the main mediators of life-history trade-offs [2,3]. Most of the ROS present in cells are produced within the mitochondria as natural by-products of aerobic respiration [1]. This has led to the pervasive idea that increased energy expenditure towards one life-history trait will result in greater ROS production, leading to accelerated senescence [4–6]. However, it is still unclear whether higher aerobic respiration actually alters *in vivo* ROS levels [7,8].

Some of the oxygen consumed by the mitochondria (mtVO_2_) is subsequently reduced to superoxide and other ROS such as hydrogen peroxide (H_2_O_2_) and hydroxyl radicals [1]. Despite the long-held belief that individual organisms consuming more oxygen have higher ROS production, the relationship between mtVO_2_ and *in vitro* ROS production is unclear, with studies reporting positive, negative or no correlation between mtVO_2_ and ROS among individuals [8]. However, several recent studies have raised reservations over the measurement of ROS production *in vitro* [8–10], since artificially high levels of metabolic substrates [11] and very high partial pressures of oxygen (20% in contrast to approx. 5% *in vivo* [10]) make extrapolations of *in vitro* results to the *in vivo* situation potentially problematic [7,8]. This is especially true when examining among-individual variation in mitochondrial traits, given that the *in vitro* conditions standardize the mitochondrial environment among individuals, which may hide the actual sources of variability in the relationship between oxygen consumption *in vivo* and mitochondrial ROS production [11].

To avoid some of these potential confounding effects *in vitro*, a ratiometric probe called MitoB has recently been developed to infer the level of mitochondrial H_2_O_2_ *in vivo* [12]. When this artificial probe compound is administered to the organism, it becomes concentrated within the mitochondria, where it is converted to its alternative form MitoP by H_2_O_2_. The level of mitochondrial H_2_O_2_ can then be expressed as the rate at which MitoB is converted to MitoP [12]. Here, we use the MitoB probe to directly quantify variation in H_2_O_2_ content in living animals and relate this to variation in whole animal oxygen consumption among individuals of similar age and under identical environmental and nutritional states, using brown trout (*Salmo trutta*), a species known to exhibit consistent individual differences in oxygen consumption [13].

## 2. Material and methods

Juvenile brown trout *S. trutta* were collected from the wild and then kept in individual compartments for 22 weeks under standard conditions of temperature (mean ± actual range: 11.5 ± 1°C) and photoperiod (12 L : 12 D), as described for these same individuals in [14]. Forty fish were randomly assigned to eight batches of five and fed daily with a specific ration calculated for each fish based on its weight [15].

At week 21, the standard metabolic rate (SMR), defined as the oxygen consumption of a resting and post-absorptive ectotherm at a given temperature, was measured over a 20 h period using flow-through respirometry (further details in [14] and electronic supplementary material). The SMR was calculated as the mean of the lowest 10th percentile of oxygen consumption measurements after controlling for body mass and is described hereafter as the residual SMR (rSMR), in mg O_2_ h**^−^**^1^.

Each fish was allowed a week of recovery after its SMR measurement before being injected with 50 nmol of MitoB (initial concentration of MitoB: 5.44 ± 0.21 nmol g**^−^**^1^ of fish). The fish were then culled after 24 h, and aliquots of their liver were immediately flash frozen for subsequent extraction and quantification of the amounts of MitoB and MitoP [12]. The content in MitoP and MitoB was determined by high performance liquid chromatography-tandem mass spectrometry and used to estimate H_2_O_2_ levels as the ratio of MitoP/MitoB.

Citrate synthase (CS) and cytochrome c oxidase (COX) activities were measured to determine liver mitochondrial density [16]. We analysed the link between the rSMR and MitoP/MitoB ratio using a general linear mixed model approach. The model included the MitoP/MitoB ratio as the dependent variable and rSMR, CS, COX and initial concentration of MitoB in the fish as continuous predictors, with batch as a random effect (see the electronic supplementary material for details of all assay protocols and statistical analyses).

## 3. Results

**Figure 1.**
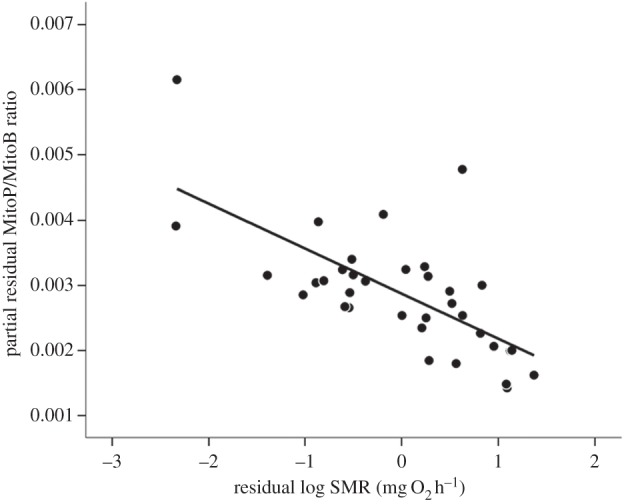
The MitoP/MitoB ratio, a proxy of *in vivo* mitochondrial H_2_O_2_ levels*,* as a function of mass-independent SMR in brown trout (*Salmo trutta*) at 12°C. Values for the MitoP/MitoB ratio are adjusted to control for the random effects of processing batch; see the electronic supplementary material for calculation of the MitoP/MitoB ratio. Solid line indicates the regression line; see text for statistical analysis.

The MitoP/MitoB ratio 24 h after injection with the MitoB probe varied up to 26-fold among individual fish (2.29 × 10^−4^–59.76 × 10**^−^**^4^). As the size of the fish at the time of injection ranged from 5.05 to 13.95 g (mean ± s.e. = 9.56 ± 0.29), the initial concentration of MitoB varied three-fold among individuals (3.59**–**9.90 nmol g**^−^**^1^), but as in a previous study [12], variation in this initial MitoB concentration did not explain the subsequent variation in the MitoP/MitoB ratio (*F*_1,25.33_ = 0.81, *p* = 0.38). However, a significant effect of the rSMR on the MitoP/MitoB ratio was observed (*F*_1,30.55_ = 12.04, *p* = 0.002; figure 1); fish with a higher rSMR had a lower MitoP/MitoB ratio compared with individuals with a lower rSMR. MitoP/MitoB ratios were independent of mitochondrial density, regardless of whether this was quantified in terms of COX activity (*F*_1,30.14_ = 1.78, *p* = 0.19) or CS activity (*F*_1,25.17_ = 0.20, *p* = 0.66).

## 4. Discussion

Measurement of ROS levels within living organisms make it possible to avoid potential biases introduced when using *in vitro* assays [17]. Development of the MitoB probe has enabled *in vivo* estimations of H_2_O_2_ levels over a period of several hours in mice, *Drosophila* and *Caenorhabditis elegans* [12]. We now demonstrate that this method can also be used successfully in fish over a 24 h period. Our findings reveal for the first time that individuals with a high SMR have a lower level of H_2_O_2_. The negative relationship between SMR and the level of H_2_O_2_ was independent of mitochondrial density estimates.

The MitoP/MitoB ratio represents the H_2_O_2_ content within the mitochondria and reflects the balance between the H_2_O_2_ generated by mitochondria during aerobic respiration and that scavenged by antioxidants such as mitochondrial glutathione peroxidase (GPx) [1,3]. Consequently, lower H_2_O_2_ levels may be attributed to a lower rate of mitochondrial H_2_O_2_ generation and/or greater antioxidant scavenging capacity [1,3]. The synthesis of GPx, an endogenous antioxidant, may be costly in term of resources [3,6]. Food intake in this experiment was limited and similar between individuals, so it is feasible that individuals with a lower H_2_O_2_ level may have allocated more resources towards antioxidant defences. Alternatively, they may have had a lower rate of ROS production. H_2_O_2_ arises in the mitochondria from the enzymatic conversion of superoxide anions that are produced by the respiratory chain. In order for the mitochondria to reduce oxygen to the superoxide anion, the mitochondrial respiratory chain must be in a highly reduced state [7,8]. Previous studies have shown that natural variation in mitochondrial function can have a significant influence on mitochondrial H_2_O_2_ production, but also on the relationship between oxygen consumption and H_2_O_2_ generation [7,8,18]. One key parameter capable of influencing such a relationship is the degree of mitochondrial uncoupling: higher uncoupling can lead to lower membrane potentials and greater rates of electron and oxygen flow in the respiratory chain (so making it less reduced) [7]. Uncoupling is known to simultaneously increase SMR and decrease ROS generation *in vitro* [7,19,20]. Our study is the first to report a negative relationship between oxygen consumption and H_2_O_2_ levels *in vivo*, but measurements of H_2_O_2_ production and H_2_O_2_ scavenging are now required in order to understand the mechanisms underlying variability in H_2_O_2_ levels.

The SMR of brown trout varies considerably among individuals of the same age and size within a population [13]; this level of interindividual variation in oxygen consumption is common across a broad range of taxa [21]. We demonstrate that a lower level of mitochondrial H_2_O_2_ is associated with a higher respiration rate, which must increase the rate at which energy substrates are oxidized. A lower H_2_O_2_ may therefore carry a cost in terms of a decrease in resources available for other traits such as body reserves or growth [2,3,6]. However, alleviation of the accumulation of oxidative damage may be a selective advantage for individual trout that have a higher rate of aerobic respiration and less H_2_O_2_ [7,19,20]. If oxidative damage is an important contributor to ageing [1], then those individuals with a high SMR may benefit through slower ageing but at a cost of reduced resource allocation to other traits, although this requires further study [22].

## Supplementary Material

Electronic Supplementary material

## Data Accessibility

Data are available from the Dryad Digital Repository: http://dx.doi.org/10.5061/dryad.t5c10.
