# Supplementary material for: Individuals with higher metabolic rates have lower levels of reactive oxygen species in vivo
Source: Biol Lett. 2015 Sep;11(9):20150538. doi: 10.1098/rsbl.2015.0538 (PMC4614431; doi:10.1098/rsbl.2015.0538)
Supplement: Electronic Supplementary material [file rsbl20150538supp1.docx]

**Individuals with higher metabolic rates have lower levels of reactive oxygen species *in vivo***

*Karine Salin^1*^, Sonya K. Auer^1^, Agata M. Rudolf^1,2^, Graeme J. Anderson^1^, Andrew G. Cairns^3,4^, William Mullen^5^, Richard C. Hartley^3^, Colin Selman^1^and Neil B. Metcalfe^1^*

^1^ Institute of Biodiversity, Animal Health & Comparative Medicine, University of Glasgow, UK.

^2^ Present address: Institute of Environmental Sciences, Jagiellonian University, Poland

^3^ School of Chemistry, University of Glasgow, UK.

^4^ Present address: Department of Chemistry, Umea University, Sweden

^5^ Institute of Cardiovascular and Medical Sciences, University of Glasgow, UK

Short title: metabolism and ROS *in vivo*

*Author for correspondence ([salin.karine@gmail.com](mailto:salin.karine@gmail.com))

Key words: fish, inter-individual variation, MitoP/MitoB ratio, oxidative stress, oxygen consumption

**ELECTRONIC SUPPLEMENTARY MATERIAL (ESM)**

**Collecting and housing.**

Juvenile brown trout collected from the wild were transported to the University of Glasgow where they were held in a 400 litre tank and allowed to acclimate for one month. Fish were then reared in individual compartments of a stream tank system under standard conditions of temperature (mean ± actual range 11.5 ± 1˚C) and photoperiod (12 L: 12 D) and food (Inicio Plus trout pellets 141, BioMar Ltd, Grangemouth, UK) (see more details in [1]). During 17 weeks of acclimation within the individual compartments, fish were individually provided with a specific food ration every day calculated as: calories = 16.3*W* ^0.737^ *e* ^(0.154^*^T^*^)^, where *W* is body mass (g) and *T* is water temperature 12˚C [2].

From week 18 to 22, the daily ration was reduced to: calories = 11.4W ^0.737^ e ^(0.154T)^ [2]. This ration was calculated to be sufficient to allow the fish to grow at a modest rate (data not shown) but was insufficient to cause satiation, so that the entire ration would be eaten each day and food intake (relative to body mass) would therefore be similar between all individuals over the 5 weeks. Fish were kept on this ration for the rest of the experiment, except during measurements of both the standard metabolic rate (S**MR)** and hydrogen peroxide (H_2_O_2_) content and 48h preceding these, on week 21 and 22 respectively. Fish were weighed every 2 weeks to adjust food rations to changes in their body size. Water quality was maintained by siphoning water and faecal matter on a daily basis. Feeding adjustments, **SMR measurements, and exposure to the MitoB probe were processed in 8 batches of 5 fish over 8 days.**

**Measurement of oxygen consumption**

**SMR** was measured in undisturbed fish in a flow through system over a 20h period at 11.5 ˚C. Full details are given in [1], but in brief, batches of five fish were kept in individual respirometry chambers in the dark, with an empty chamber being used as a control. Air-saturated water was pulled through the chambers with a peristaltic pump at 1.68 L h^-1^ to a fibre-optic oxygen sensor (robust probe; PyroScience GmbH, Aachen, Germany). Oxygen (O_2_), temperature, and barometric pressure data were read by multichannel oxygen meters (FireStingO2, PyroScience) and collected using FireSting software version 3.0 (PyroScience). Oxygen consumption (mg O_2_ h^-1^) was calculated according to the equation

***M*_O2_ = *V*_w_ × (*C*_wO2control_ - *C*_wO2fish_)**,

where V_w_ is the flow rate of water through the respirometry chamber (L h^-1^), and C_wO2control_ and C_wO2fish_ are the concentrations of oxygen (mg L^-1^) in the outflow of the chambers lacking and containing fish respectively, after adjusting for temperature and barometric pressure. **SMR** was calculated by taking the mean of the lowest 10^th^ percentile of oxygen consumption measurements over the 20h measurement period, and then excluding outliers, i.e. those measurements below 2 standard deviations from this mean [3]; SMR was expressed as mg O_2_ h^-1^. Following the **SMR** measurement, body weight was measured by briefly anaesthetising the fish (50 mg/l benzocaine diluted in water). Fish were then returned to their individual tanks and allowed a week of recovery. The SMR of juvenile brown trout kept under constant condition has been shown to be highly repeatable over periods of several weeks [4, 5]. Consequently, SMR at week 22, when the H_2_O_2_ assay was measured, was likely to be consistent with SMR measured at week 21.

**Fish exposure to the MitoB probe**

**Measurements of *in vivo*** H_2_O_2_ **levels were made using the MitoB probe adapted for use on fish from the protocol of Cocheme *et al*. [6].** Fish were transferred at week 22 to individual tanks containing 2 L of aerated water and allowed to acclimate without food and in darkness for 24h. They were then briefly anaesthetised (50 mg/ml benzocaine diluted in water) and given an intraperitoneal injection of a standard dose of MitoB solution (100 µL of 504.09 µM MitoB, i.e. 50 nmol/fish), previously diluted in 0.7% (v/v) ethanol and sterile saline solution 0.9% (w/v) NaCl / H_2_O. Each fish was returned to its individual tank and left for at least 24h (mean ± SE: 27.95 ± 0.10h, **no detectable effect of this** duration on **H_2_O_2_ content**: F_1, 30.74_ = 0.73, *P* = 0.40) without food and in darkness to simulate the conditions under which SMR was measured. The fish were then culled and their liver immediately dissected and flash frozen in liquid nitrogen. Liver was chosen because previous studies have shown a strong association between SMR and hepatic mitochondrial function [7, 8]. A supplementary group of 8 control fish from the same cohort of brown trout collected from the wild was treated in exactly the same manner as the experimental fish except they were not anaesthetised nor injected with MitoB solution. One control fish was included per batch processing in order to obtain a blank control value for the subsequent high performance liquid chromatography-tandem mass spectrophotometer (HPLC-MS) analysis. The time course of the compounds MitoB and MitoP in the fish was assessed in an independent group of juvenile brown trout kept at 12˚C. The amount of MitoB (injection of 50 nmol/fish, n = 20 fish) or MitoP (injection of 50 nmol/fish, n = 20 fish) per a mg of liver was recorded 3h, 12h, 24h, 48h and 72h post-injection (n = 4 fish per time point). There was no detectable difference in the kinetic between MitoB and MitoP (mean ± SE: MitoB = -0.20 ± 0.45 pmol h^-1^ mg liver^-1^; MitoP = -0.09 ± 0.45 pmol h^-1^ mg liver^-1^; F_1, 77.02_ = 0.03, *P* = 0.86).

**Extraction of the probes**

**The MitoB and MitoP probes and their deuterium spikes** deuterium_15_-MitoB (***d_15_*MitoB**) and deuterium_15_-MitoP (***d_15_*MitoP**) **were synthesized at the University of Glasgow according to [9] and kept at -20 °C. Methods for extraction, quantification and analysis of the probe were adapted from [6]. Extractions were processed in 4 batches, each containing two blank control fish plus 10 experimental fish (combining 2 of the batches of five fish processed for the measurement of SMR and exposure to the probe). To extract MitoB and MitoP from fish livers, aliquots (**mean ± SE: 15.43 ± 0.73 mg) were homogenised in 500 µL of a solution containing 60% (v/v) acetonitrile (ACN) and 0.1 % (v/v) formic acid (FA), using a Potter tissue grinder (cordless motor, Sigma-Aldrich) with a Teflon pestle in a 1.5 mL Eppendorf tube on ice. The pestle was washed with 250 µL of the solution of 60% ACN and 0.1 % v/v FA and this wash was combined with the original homogenate. Homogenates were spiked with 10 µL of absolute ethanol containing 100 pmol ***d_15_*MitoB** and 50 pmol ***d_15_*MitoP** and vortexed for 30 sec. After centrifuging for 10 min at 16,000 g, the supernatant was keep aside and the pellet re-extracted by vortexing in 500 µL of the solution of 60% ACN, 0.1 % FA. After further centrifuging for 10 min at 16,000 g, the second supernatant was pooled with the first one, and centrifuged for a final 10 min at 16,000g in microcentrifuge filters (containing a PVDF membrane with pore size of 0.22 µm between the upper and lower parts of the tube). The filtered solution was collected in the lower part of the microcentrifuge filter and transferred into a new tube to dry under a vacuum overnight. The dried samples were re-dissolved in 200 µL of a solution containing 20% ACN, 0.1 % FA by vortexing for 30 sec. These dissolved samples were then centrifuged for 10 min at 16,000g and the supernatant carefully transferred to auto-sampler vials, which were stored at -70˚C until HPLC-MS analysis for quantification of relative concentrations of the two versions of the probe and their spikes.

**Quantification of the probes**

Analysis of the **MitoB, MitoP, *d_15_*MitoB** and ***d_15_*MitoP** was conducted using a Thermo Scientific Accela™ HPLC (Thermo Fisher Scientific, San Jose, CA, USA), consisting of an HPLC pump, and an autosampler cooled to 4˚C. Chromatographic separation **was performed at 40˚C** using a hypersil gold column (150 mm x 2 mm, I.D. 2.0µm) with guard column at **constant flow rate of 300 µL min^-1^. The mobile phase comprised** ACN and 0.1 % FA, delivered according to the **gradient program given** in table S1. Fish samples or standards (10 µL) were injected in the mobile phase. After passing through the HPLC, the column eluate was directed to an Exactive Orbitrap trap MS (Thermo Fisher Scientific, Hemel, UK) fitted with an electrospray interface in order to quantify the peak. **Nitrogen was used as the sheath and auxiliary gas, with electrospray ionization in positive ion mode (sheath and auxiliary gas = 60 and 20 units; spray voltage = 4.5 kV; capillary temperature = 300 ˚C).** The mass spectrophotometer was set up in positive ionization to scan from **mass-to-charge ratio *(m/z)* 350.00 to *m/z* 420.00, resolution was set at 60,000. The HPLC-MS system was controlled using Xcalibur software (Thermo Fisher Scientific).**

**Calibration curves were performed with standards of MitoB, *d_15_*MitoB, MitoP and *d_15_*MitoP (Table S2).** Vials containing samples (n = 40 treated plus 8 control fish, diluted by 10) and standards (n = 10) were defrosted, vortexed for few seconds, and placed in an autosampler in a refrigerated holder at 4 ˚C until the end of the analysis. **All vials were run in duplicate (**repeatability **of measurements, all based on n = 58 samples, MitoP:** r = 0.98, *P* < 0.001**, *d_15_*MitoP**: r = 0.75, *P* < 0.001, **MitoB:** r = 0.98, *P* < 0.001**, *d_15_*MitoB:** r = 0.81, *P* < 0.001).

**Calculation of MitoP/MitoB ratio**

**For all analysed samples (control fish, treated fish and standards), the absolute area for the 4 peaks (i.e. for MitoP, *d_15_*MitoP, MitoB and *d_15_*MitoB) from the HPLC-MS response was analysed by Xcalibur software (Thermo Fisher Scientific) according to their mass-to-charge ratios *(m/z)*, within *m/z* 369.1380-369.1440, *m/z* 384.2310-384.2370, *m/z* 397.1500-397.1560 and *m/z* 412.2440-412.2500 respectively. Peaks from the standards were used to produce calibration curves of mean of absolute area of the two replicates against pmol of MitoP, *d_15_*MitoP, MitoB and *d_15_*MitoB (Figure S1); Absolute areas for the treated samples lay within the linear section of the calibration curves, except for two samples which were excluded from subsequent analyses (descriptive statistics given in table S3). The absolute areas of MitoP and MitoB for the control fish were below the detection level and so were not considered for calculation. The mean absolute areas from the two analysed samples per treated fish were determined for each of the four compounds, and converted into pmol using the appropriate calibration curve.**

**The homogenates initially contained** 100 pmol ***d_15_*MitoB** and 50 pmol ***d_15_*MitoP** as internal spikes, which allowed calculation of individual coefficients for extraction efficiency of ***d_15_*MitoB** and ***d_15_*MitoP** from the final extracted values (coefficient of extraction ***d_15_***Mito B = 0.39 ± 0.02, ***d_15_***Mito P = 0.42 ± 0.04). The amounts of **MitoP and Mito B were corrected using these individual extraction coefficients, and the ratio MitoP/MitoB calculated for each fish. Finally**, mitochondrial H_2_O_2_ content was estimated as the MitoP/MitoB ratio using the equation below:

$$MitoP/MitoB ratio = \frac{1}{t}ln\left( \frac{MitoP}{MitoB}+1 \right)$$

where *t* = actual duration of MitoB exposure in hours. The ratio MitoP/MitoB is a suitable proxy of H_2_O_2_ content even if difference in membrane potential among individuals occurred because the amount of MitoB that accumulates in the mitochondrial matrix is relatively consistent over the range of membrane potential *in vivo* [10].

**Citrate synthase and Cytochrome c oxidase activity**

The citrate synthase (CS) and cytochrome c oxidase (COX) assays were adapted from [11]. Frozen liver was homogenized with a Potter tissue grinder with a Teflon pestle in a 1.5 mL Eppendorf tube on ice at a concentration of 100 mg wet tissue / mL buffer (20 mM Tris, 100 mM KCl, 2 mM EGTA, 250 mM Sucrose and pH 7.4 at 4°C). The homogenate was centrifuged at 600g for 10min at 4°C, and then 3 aliquots of supernatant (one for each of protein assay, CS assay and COX assay, so ensuring no repetition of freezing - thawing step) were flash frozen in liquid nitrogen and stored at -70°C until analysed.

Determination of the protein content of the liver was performed in duplicate using a bicinchoninic acid assay [12], where bovine serum albumin was used as standard and absorbance was read at 562 nm (repeatability: n = 35, r = 0.98, *P* < 0.001).

Enzymatic activities were measured spectrophotometrically at 25°C, in triplicate on a microplate (POLARstar® Omega, BMG labtech) and with shaking. COX activity was assessed in KPi buffer (50 mM, pH 7.4) pre-equilibrated at 25°C. Reduced cytochrome c was prepared from equine heart cytochrome c solution incubated for 1 hour with ascorbic acid solution at 4°C (pH 7.0). The mixture was filtered in a desalting column to purify the reduced cytochrome c solution from the ascorbic acid. Aliquots of reduced cytochrome c solution were kept in liquid nitrogen until use. The stability of the solution was tested before each COX activity assay (see below for details as same condition as the blank activity): the decrement of a 50 µM solution at 550 nm was below 0.005 OD per minute, indicating no excessive auto-oxidation [11].

Before each COX activity assay, the concentration of the reduced cytochrome c solution was measured at 550 nm and its state of reduction was assessed by calculating the ratio of the absorbance values at 550 nm to those at 565 nm, with a ratio greater than 6 being considered acceptable. The absorbance of the blank was measured in the presence of 50 µM of reduced cytochrome c, and monitored as the rate of disappearance of reduced cytochrome c by measuring the absorbance at 550 nm every 15 sec for 3 min; this rate was found to be linear over the last min. The COX activity of the sample was initiated by adding 12.20 mg mL^-1^ of sample to the solution of 50 µM of reduced cytochrome c, and immediately measuring the absorbance at 550 nm every 15 sec for 1 min. Background activity was evaluated for 20 of the 40 samples by running the assay in parallel with 300 µM of potassium cyanide; it was found not to be detectable, so no correction was needed. The results were expressed as the rates of reduced cytochrome c disappearance (nmol min^-1^ mg^-1^ of protein) using an extinction coefficient of 18.5 (repeatability of triplicate: n = 34, r = 0.85, *P* < 0.001).

CS activity was assessed using a homogenate concentration of 2.5mg mL^-1^ of a Tris buffer (100 mM Tris, pH 8, 0.1 % (v/v) triton 100X) pre-equilibrated at 25°C. CS activity was monitored as the rate of generation of thionitrobenzoate anion, by measuring the absorbance at 412 nm. For each replicate blank activity levels were calculated by running the homogenate in buffer with 100µM 5,5′-dithiobis(2-nitrobenzoic acid) and 300µM acetyl CoA for 1 min. The reaction was then initiated by adding 0.1 mM oxaloacetic acid and measuring the absorbance for 10 min. The results were expressed as the rate of production of thionitrobenzoic acid (nmol min^-1^ mg^-1^ of protein) using an extinction coefficient of 13.6 (repeatability of triplicate: n = 31, r = 0.50, *P* < 0.05).

**Statistical analyses**

**SMR and body mass were log_10_-transformed. We used regression analysis of log SMR on log body mass to determine the residual variation in SMR (rSMR) once the effects of body mass were removed (Figure S2). We analysed the factors predicting inter-individual variation in** MitoP/MitoB ratio **using a linear mixed effect model, with** MitoP/MitoB ratio **as the dependent variable, rSMR, CS activity, COX activity and concentration of MitoB at injection as continuous predictors, and batch processing as a random effect. Non-significant terms were dropped sequentially until only significant factors remained. Collinearity diagnostics were performed by quantifying variance inflation factors (VIF) to explore multi-collinearity among the predictor variables, but VIF values were all found to be below 2 indicating that there were no issues of collinearity.** Two fish had outlier values for one of the Mito compounds and were dropped from the analysis, so that analyses are based on a sample size of 38 fish, with the exception of those analyses including COX and CS activity where the sample size was 35 because COX and CS activity was not measured in 3 individuals. **Data were analysed using IBM SPSS Statistics 22 (SPSS Inc., Chicago, IL, USA). T**he level of significance was set to *P* < 0.05.

**References**

[1] Auer, SK, Salin, K, Rudolf, AM, Anderson, GJ, Metcalfe, NB (2015) The optimal combination of standard metabolic rate and aerobic scope for somatic growth depends on food availability. *Functional Ecology* **29**, 479-486.

[2] Elliott, J.M. 1976 The energetics of feeding, metabolism and growth of brown trout (*Salmo trutta L.*) in relation to body weight, water temperature and ration size. *Journal of Animal Ecology* **45**, 923-948. (doi:10.2307/3590).

[3] Clark, T.D., Sandblom, E. & Jutfelt, F. 2013 Aerobic scope measurements of fishes in an era of climate change: respirometry, relevance and recommendations. *The Journal of Experimental Biology* **216**, 2771-2782.

[4] Norin, T. & Malte, H. 2011 Repeatability of standard metabolic rate, active metabolic rate and aerobic scope in young brown trout during a period of moderate food availability. *The Journal of Experimental Biology* **214**, 1668-1675. (doi:10.1242/jeb.054205).

[5] Auer, S.K., Salin, K., Rudolf, A.M., Anderson, G.J. & Metcalfe, N.B. 2015 Flexibility in metabolic rate confers a growth advantage under changing food availability. *Journal of Animal Ecology*, n/a-n/a. (doi:10.1111/1365-2656.12384).

[6] Cochemé, H.M., Logan, A., Prime, T.A., Abakumova, I., Quin, C., McQuaker, S.J., Patel, J.V., Fearnley, I.M., James, A.M., Porteous, C.M., et al. 2012 Using the mitochondria-targeted ratiometric mass spectrometry probe MitoB to measure H_2_O_2_ in living Drosophila. *Nature Protocols* **7**, 946-958. (doi:10.1038/nprot.2012.035).

[7] Brookes, P.S., Buckingham, J.A., Tenreiro, A.M., Hulbert, A.J. & Brand, M.D. 1998 The proton permeability of the inner membrane of liver mitochondria from ectothermic and endothermic vertebrates and from obese rats: correlations with standard metabolic rate and phospholipid fatty acid composition. *Comparative Biochemistry and Physiology B-Biochemistry & Molecular Biology* **119**, 325-334.

[8] Brown, J.C.L., Chung, D.J., Belgrave, K.R. & Staples, J.F. 2012 Mitochondrial metabolic suppression and reactive oxygen species production in liver and skeletal muscle of hibernating thirteen-lined ground squirrels. *Am. J. Physiol.-Regulat. Integr. Compar. Physiol.* **302**, R15-R28. (doi:10.1152/ajpregu.00230.2011).

[9] Cairns, A., McQuaker, S., Murphy, M. & Hartley, R. 2015 Targeting mitochondria with small molecules: The preparation of mitob and mitop as exomarkers of mitochondrial hydrogen peroxide. In *Mitochondrial Medicine* (eds. V. Weissig & M. Edeas), pp. 25-50, Springer New York.

[10] Cochemé, H.M., Quin, C., McQuaker, S.J., Cabreiro, F., Logan, A., Prime, T.A., Abakumova, I., Patel, J.V., Fearnley, I.M., James, A.M., et al. 2011 Measurement of H_2_O_2_ within living drosophila during aging using a ratiometric mass spectrometry probe targeted to the mitochondrial matrix. *Cell Metabolism* **13**, 340-350. (doi:10.1016/j.cmet.2011.02.003).

[11] Spinazzi, M., Casarin, A., Pertegato, V., Salviati, L. & Angelini, C. 2012 Assessment of mitochondrial respiratory chain enzymatic activities on tissues and cultured cells. *Nature Protocols* **7**, 1235-1246. (doi:10.1038/nprot.2012.058).

[12] Smith, P.K., Krohn, R.I., Hermanson, G.T., Mallia, A.K., Gartner, F.H., Provenzano, M.D., Fujimoto, E.K., Goeke, N.M., Olson, B.J. & Klenk, D.C. 1985 Measurement of protein using bicinchoninic acid. *Analytical Biochemistry* **150**, 76-85. (doi:<http://dx.doi.org/10.1016/0003-2697(85)90442-7)>.

**Figure S1**: Calibration curves for (A) **MitoP, (B) *d_15_*MitoP, (C) MitoB and (D) *d_15_*MitoB detection by HPLC-MS. Each point is the mean ± SE of duplicate measurements. Inserts show the data points lying near the origin of the graph. The calibration curves were generated from the standards that had an absolute area within the detection limits of the HPLC-MS, *i.e.* those standards with the lowest and highest probe concentrations were excluded from the calculation of the calibration curve.**

**A.**

B.

C.

D.

**Figure S2**: Relationship between standard metabolic rate (**SMR**) and body mass of brown trout *Salmo trutta*. **log_10_ SMR as a function of log_10_ body mass where log_10_ SMR = 0.874 ± 0.171 log_10_ BodyMass + 1.875 ± 0.167 (*r^2^* = 0.42, *P*<0.001); residuals from this relationship (rSMR values) were used in subsequent analyses.**


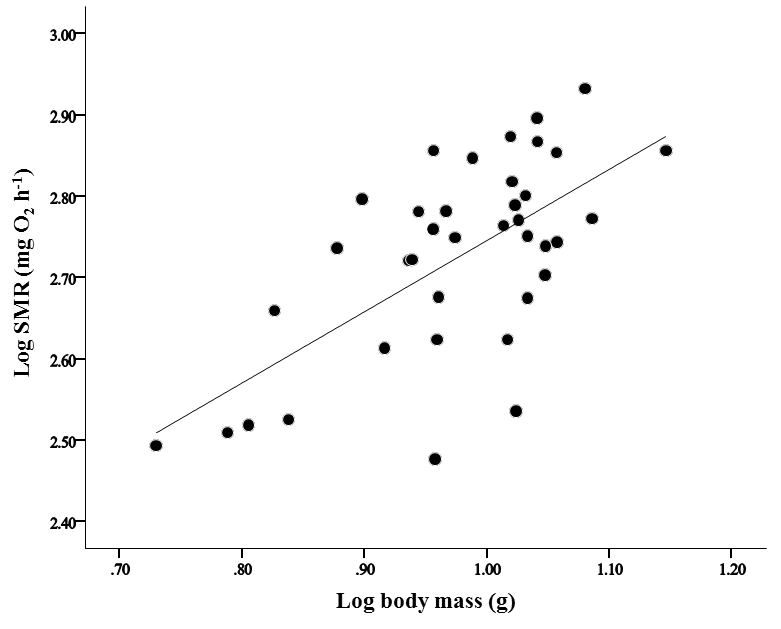
 **Table S1**: Delivery sequence **of the gradient elution in the HPLC-MS analysis, with a mobile phase containing** acetonitrile (ACN) and 0.1% formic acid (FA) under a constant flow rate of 300 µL min^-1^

| Time (min) | FA (%) | ACN (%) |
| --- | --- | --- |
| 0.00-9.90 | 70.0 | 30.0 |
| 9.90-10.00 | 30.0 | 70.0 |
| 10.00-13.10 | 5.0 | 95.0 |
| 13.10-17.00 | 70.0 | 30.0 |

**Table S2**: Amount of the probes in the 10 standards used to generate **MitoP, *d_15_*MitoP, MitoB and *d_15_*MitoB** calibration curves.

| Standard | ProbeP (pmol) | *d_15_*ProbeP (pmol) | ProbeB (pmol) | *d_15_*Probe B (pmol) |
| --- | --- | --- | --- | --- |
| 1 | 1313 | 100 | 5040 | 200 |
| 2 | 656.5 | 50 | 2520 | 100 |
| 3 | 131.3 | 10 | 504 | 20 |
| 4 | 65.65 | 5 | 252 | 10 |
| 5 | 13.13 | 1 | 50.4 | 2 |
| 6 | 6.565 | 0.5 | 25.2 | 1 |
| 7 | 1.313 | 0.1 | 5.04 | 0.2 |
| 8 | 0.6565 | 0.05 | 2.52 | 0.1 |
| 9 | 0.32825 | 0.025 | 1.26 | 0.05 |
| 10 | 0 | 0 | 0 | 0 |

**Table S3**: Descriptive statistics of the absolute areas obtained from the HPLC-MS runs for samples from the treated fish. Statistics are shown for the duplicate 1 and 2 of each of the 4 probes. Note that the sample size is reduced to 38 because one sample was excluded for showing an absolute area for *d_15_*MitoP above the standard range, and another one for showing a value for MitoP below the standard range.

|  | N | Minimum | Maximum | Mean | SE |
| --- | --- | --- | --- | --- | --- |
| **MitoP - 1** | 38 | 3.60 10^4^ | 1.37 10^7^ | 1.10 10^6^ | 4.21 10^5^ |
| **MitoP - 2** | 38 | 4.24 10^4^ | 4.79 10^6^ | 5.76 10^5^ | 1.47 10^5^ |
| ***d_15_*MitoP - 1** | 38 | 2.73 10^5^ | 4.55 10^6^ | 1.52 10^6^ | 1.87 10^5^ |
| ***d_15_*MitoP - 2** | 38 | 4.72 10^5^ | 1.53 10^6^ | 8.23 10^5^ | 3.57 10^4^ |
| **MitoB - 1** | 38 | 1.23 10^6^ | 3.74 10^7^ | 1.22 10^7^ | 1.49 10^6^ |
| **MitoB - 2** | 38 | 1.14 10^6^ | 2.01 10^7^ | 7.12 10^6^ | 5.74 10^5^ |
| ***d_15_*MitoB - 1** | 38 | 4.30 10^5^ | 5.10 10^6^ | 2.32 10^6^ | 2.21 10^5^ |
| ***d_15_*MitoB - 2** | 38 | 7.88 10^5^ | 1.71 10^6^ | 1.30 10^6^ | 3.28 10^4^ |
